# Peer Review of “Risk Factors of SARS-CoV-2 Infection: Global Epidemiological Study”

**DOI:** 10.2196/31927

**Published:** 2021-08-26

**Authors:** Palash Banik

**Affiliations:** 1 Department of Noncommunicable Diseases Bangladesh University of Health Sciences Darus Salam Bangladesh

*This is a peer-review report submitted for the paper "Risk Factors of SARS-CoV-2 Infection: Global Epidemiological Study".*

## Round 1 Review

### General comments

This paper [1] is nicely written by the author, and it is indeed a very important work on SARS-CoV-2 infection. The author presents all the data appropriately and discusses the data rationally. It will be a very interesting and vital reference paper to all those who are working on this topic. I want to see the paper published soon. Best wishes.
